# His Bundle pacing for congenital complete AV block: An attempt to fix a broken heart?

**DOI:** 10.1111/anec.12895

**Published:** 2022-03-02

**Authors:** Giuseppe Pio Piemontese, Matteo Ziacchi, Giovanni Statuto, Andrea Angeletti, Giulia Massaro, Lorenzo Bartoli, Mauro Biffi

**Affiliations:** ^1^ Department of Experimental, Diagnostic and Specialty Medicine Institute of Cardiology IRCCS Azienda Ospedaliero‐Universitaria di Bologna Bologna Italy

**Keywords:** congenital atrioventricular block, his bundle pacing, left ventricular dysfunction, ventricular dyssynchrony

## Abstract

Congenital complete atrioventricular block (CCAVB) is usually due to failure of atrioventricular nodal conduction with preservation of the His‐Purkinje system. Most patients with CCAVB ultimately require pacemaker therapy to restore physiologic heart rates, dealing with the detrimental effects of chronic right ventricular (RV) pacing on cardiac structure and function. The ideal stimulation pattern aims to mimic the normal conduction to restore electromechanical coupling, preventing the harmful effects of lack of atrioventricular and inter‐intraventricular synchrony. This can be done through conduction system pacing. Using His bundle pacing (HBP) for cardiac resynchronization therapy in two complete congenital atrioventricular block patients, we have reported better exercise tolerance and echocardiographic improvements related to reversible left ventricular dysfunction that can be corrected by restoration of the normal activation pathway via the His‐Purkinje network.

## 1 BACKGROUND

The incidence of congenital complete atrioventricular block (CCAVB) is 0.5–1/15.000 births (Michaëlsson et al., 1995) and is due to failure of atrioventricular (AV) nodal conduction with preservation of the His‐Purkinje system.

The implantation of a pacemaker is recommended for symptomatic patients and for asymptomatic patients with ventricular dysfunction or at risk of syncope and sudden death; nonetheless, right ventricular (RV) pacing can have detrimental effects on cardiac function (Kim et al., 2007; Moak et al., 2006; Thambo et al., 2004). Left ventricular (LV) remodeling can occur and be associated with exercise intolerance/heart failure in up to 20% of adult patients (Khurshid et al., 2014), congestive heart failure being observed in 7%–10% of patients paced because of CCAVB (Kim et al., 2007; Moak et al., 2006).

While RV pacing–associated cardiomyopathy benefits from cardiac resynchronization therapy (CRT), its indication is less clear in pediatric than in older patients, owing to the low prevalence of dilated cardiomyopathy (Moak et al., 2006). Since the His‐Purkinje system is preserved in CCAVB patients, it can be expected that His bundle pacing (HBP) would be a suitable treatment for CCAVB patients with RV pacing–associated LV dysfunction, and could become the gold standard for CCAVB in the future.

## 2 CASE REPORTS

### 2.1 Case 1

**FIGURE 1:**
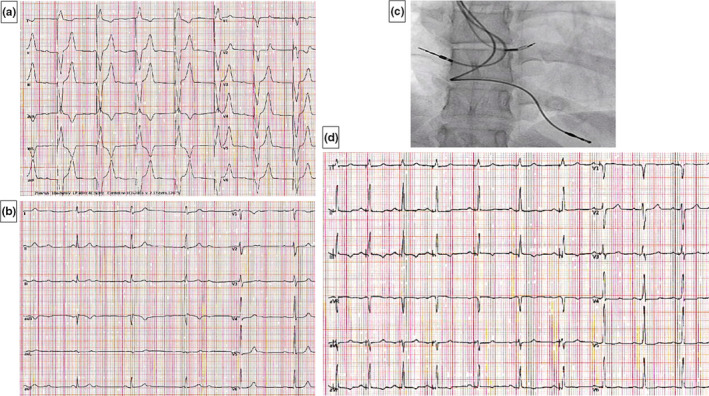
Case 1. Panel A. 12‐lead ECG showing RV pacing in DDD mode. Panel B. 12‐lead ECG showing the intrinsic junctional rhythm. Panel C. Fluoroscopic view of HBP leads during implantation. Panel D. 12‐lead ECG showing non‐selective HBP at 12 months

An 18‐year‐old girl with a history of CCAVB had a VVIR pacemaker implanted at 9. Elective replacement was indicated after 9 years of 90% VVIR pacing; echocardiographic evaluation showed slightly increased LV volume (LVEDVi = 86 ml/m^2^ and LVESVi = 50 ml/m^2^) with a mildly depressed LVEF (42%) and moderate mitral and tricuspid regurgitation. The 12‐lead ECG showed sinus rhythm with complete atrioventricular block and a ventricular‐paced QRS (duration 166 ms; Figure 1, panel A), while the intrinsic junctional rhythm at 59 bpm had a QRS duration of 82 ms (Figure 1, panel B); in need to open the pocket for device replacement, we planned an upgrade to triple‐chamber pacemaker with HBP to correct electromechanical dyssynchrony by restoring the physiologic atrioventricular, interventricular, and intraventricular synchronicity. An active‐fixation atrial lead was advanced in the right atrium, and a SelectSecure 3830 pacing lead was delivered by a Medtronic C315His catheter (Medtronic Inc) (Figure 1, panel C). Non‐selective HBP was achieved at 1.5 V@1.0 ms pacing threshold. The 3 leads were connected to a Serena CRT‐P (Medtronic Inc); SelectSecure was connected into the LV port. Atrioventricular physiologic pacing (DDD, lower rate 40 bpm and upper rate 170 bpm) with non‐selective HBP was programmed in LV‐only mode, with a QRS duration and morphology identical to the native QRS at a sensed AV interval of 100 ms (Figure 1, panel D). At 12‐month follow‐up, the HBP threshold was stable (1.5 V@1.0 ms), with 100% of pacing in DDD mode. Echocardiography showed reverse remodeling: LVEDVi = 65 ml/m^2^, LVESVi = 32 ml/m^2^, LVEF = 55%, mitral and tricuspid regurgitation decreased to mild.

### 2.2 Case 2

**FIGURE 2:**
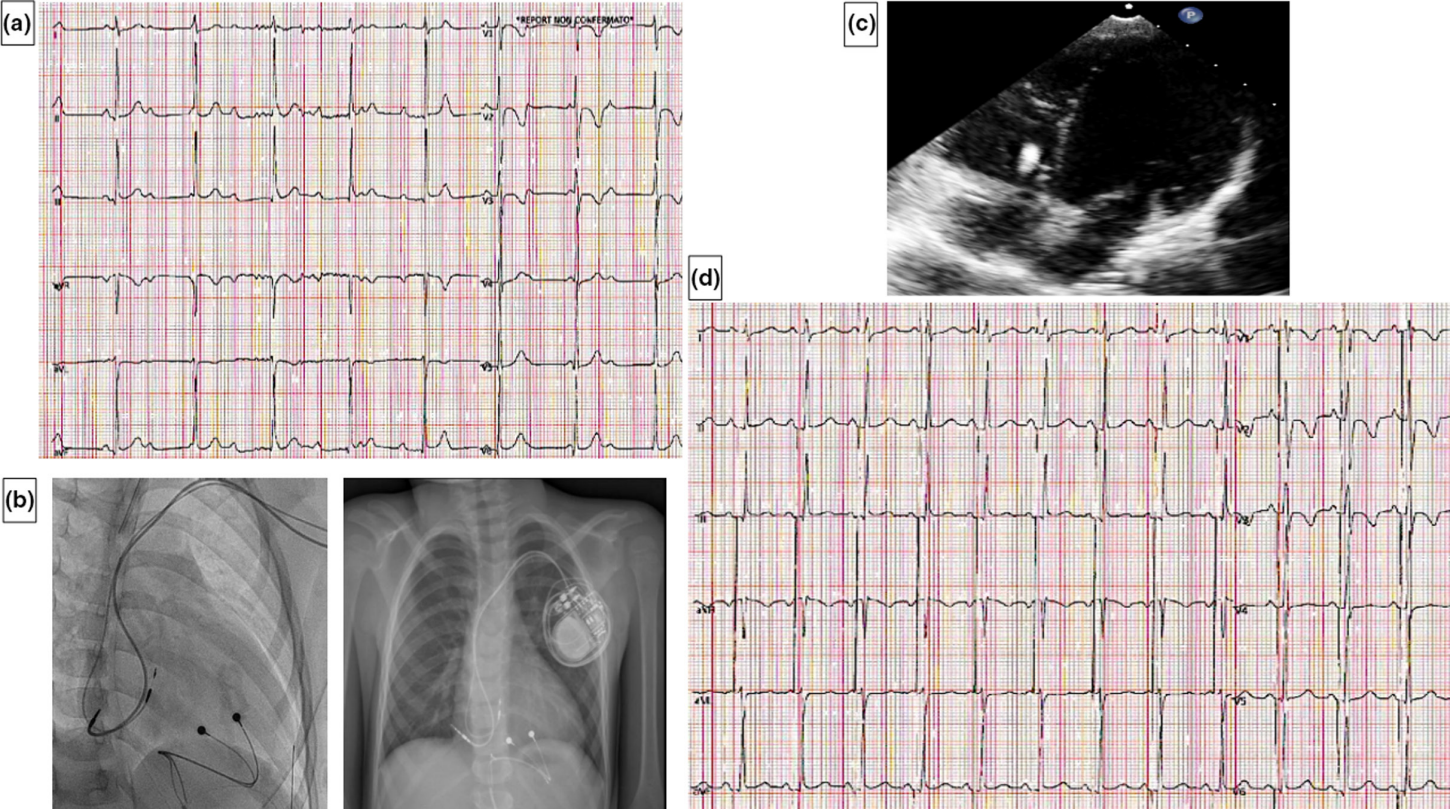
Case 2. Panel A. 12‐lead ECG showing the intrinsic junctional rhythm. Panel B. Right anterior oblique (on the left) and anteroposterior radiological view (on the right) of atrial and HBP leads after implantation. Panel C. Echocardiographic view of RV pacing‐associated cardiomyopathy with mild increase in LV volume; HBP was placed a few millimeters below the tricuspid valve. Panel D. 12‐lead ECG showing selective HBP at 12 months

A 4‐year‐old child, weighing 18 kg, was implanted when he was 10 months old with an epicardial VVIR pacemaker due to CCAVB; the pocket for pulse generator was placed in abdomen. Elective replacement was programmed after only 3 years, following a rapid consumption of the battery due to an increase in the stimulation threshold with intermittent exit block at 7.5 V. The intrinsic junctional rhythm at 33 bpm had 104 ms QRS duration (Figure 2, panel A). Echocardiography showed normal LVEF (60%) with mild increase in LV volumes (LVEDVi = 80 ml/m^2^; LVESVi = 33 ml/m^2^) associated with evident septal dyskinesia (Figure 2 panel C). Using a double left subclavian vein access, a SelectSecure 3830 pacing lead (Medtronic Inc) was used to obtain a selective HBP capture with 1,7 V@0.8 ms and an atrial lead was placed in the middle lateral wall moving along a craniocaudal approach. Both leads were given the maximum possible slack intended for patient's growth and each of the two rests on the floor of the atrium running in opposite directions (Figure 2, panel B); after creating a left subclavicular pocket, they were connected to Azure S PM (Medtronic Inc), programmed in DDD mode (Figure 2, panel D). At 12‐month follow‐up, a normalization of cardiac volumes and stability of the capture threshold have been observed.

## 3 DISCUSSION

Our first adolescent patient had an upgrade at her first pacemaker replacement after 9 years of RV pacing, exhibiting improvement of LV function and exercise tolerance following restoration of AV synchrony and of ventricular activation via the His‐Purkinje network. The same results were obtained in the second case, a child with CCAVB and early signs of reduced contractile function of the LV, which represents a successful HBP procedure in the youngest child reported to date.

CCAVB in patients with a normal heart disrupts 2 of the 3 electrophysiological determinants of cardiac performance: atrioventricular synchrony and chronotropic response. Through CCAVB patients can have a normal physical development and, occasionally, a normal life expectancy, the majority have signs of LV dysfunction, atrial arrhythmias, or symptomatic heart failure at long‐term follow‐up. When a pacing indication ensues, chronotropic response is restored at the expense of the loss of inter‐intraventricular synchrony, with or without restoration of AV synchrony depending on pacing mode (DDD vs VVIR).

In this perspective, key questions in CCAVB are yet unanswered owing to the impossibility to run methodologically correct studies in this pediatric population. The timing to consider pacing is individually based. The choice of the pacing site and mode has been mostly debated in literature (Cabrera Ortega et al., 2017; Thambo et al., 2004; Ventura et al., 2000. Despite convincing evidence of superior cardiac performance and exercise tolerance with AV‐synchronous pacing, VVIR mode seems a reasonable choice as the initial pacing strategy in small children to minimize intravascular hardware (risk of vein thrombosis) and lead malfunction, given the modest difference in clinical endpoints and quality of life in pre‐adolescence (Horenstein & Karpawich, 2004).

A first pacemaker implant or replacement after early childhood or in adolescence is a different setting, because key decisions are taken for long‐term cardiac pacing in a view to restore—possibly—all the 3 electrophysiologic determinants of cardiac function. A mild LV systolic dysfunction, especially when associated with functional mitral or tricuspid regurgitation and initial left atrial enlargement, is a clinical hint of the RV pacing detrimental effect and may promote upgrading the system to restore cardiac synchronicity. CRT is well known to improve cardiac function in RV pacing‐induced heart failure/LV dysfunction (Höijer et al., 2006); recent evidence, however, points toward a similar effect of HBP and CRT in this setting, in terms of both LV function and clinical status improvement (Vijayaraman et al., 2019). These results are not surprising, given the comparable efficacy reported for HBP and CRT in heart failure patients, with a possible beneficial effect of HBP also in non‐responders to conventional CRT (Lustgarten et al., 2015; Zanon et al., 2018).

A single case of cardiac resynchronization via direct HBP in a CCAVB patient with RV pacing‐associated cardiomyopathy has been reported to date (Rehwinkel et al., 2011), highlighting the concept of reversible LV dysfunction that can be corrected by restoration of the normal activation pathway via the His‐Purkinje network. Our experience with milder degrees of LV dysfunction suggests a role for the preservation of LV mechanics at long term in CCAVB with persistent His‐to‐ventricle conduction, when the first replacement after early childhood or in adolescence offers the opportunity to upgrade the pacing system. Dandamudi et al. have recently reported on 17 CCAVB patients aged 27 ± 11 years, eight of which previously RV‐paced. Improvement of ventricular function was observed in 3 patients in RV pacing‐induced LV dysfunction; 2/17 (11.7%) patients required HBP lead revision due to elevated pacing thresholds (Dandamudi et al., 2020).

The ongoing development of dedicated tools and the improved skillfulness in HBP implantation have now set the ground for the expansion of this pacing modality to CCAVB patients.

### 3.1 Limitations

The observations about these two patients need to be confirmed in a large series of CCAVB patients. Moreover, some caveats are to be considered, such as persistence of conduction in the His‐Purkinje network at long term; minimization of intravascular leads that dwell lifelong in young patients; device longevity when a moderate‐to‐high HBP threshold dictates a high current drain; and lead revision in the event of loss of capture, as the number of pocket surgeries is a main predisposing factor to infection. Technical aspects like the amount of lead slack need to accommodate for patients’ growth are better addressed in late rather than in early childhood.

## 4 CONCLUSIONS

Taken these points as a habit at innovation, we believe that HBP should be explored as the first‐choice strategy in CCAVB undergoing pacemaker implantation in childhood, and in CCAVB candidates to upgrading because of RV pacing–associated LV dysfunction. A challenge may be represented by the possible increase in chronic pacing threshold, as also reported by Dandamudi et al. (Dandamudi et al., 2020) in 11.7% of patients. While much is still to be understood about the biologic behavior of cardiac conduction tissue at long term, further studies are needed to enhance persistence of a stable HBP threshold and of conduction in the His‐Purkinje network at long term. New power sources enabling high‐output use for >15 years would certainly lift the constraints currently posed by unanticipated pacing threshold increase in the long term in this young population (Biffi et al., 2021).

## CONFLICT OF INTEREST

Mauro Biffi has held educational activity and received honoraria as speaker's bureau for Biotronik, Boston Scientific, and Medtronic. Matteo Ziacchi has held educational activity and received honoraria as speaker's bureau for Biotronik, Boston Scientific Abbott and Medtronic. All other authors declare that they have no conflict of interests.

## 5 ETHICAL APPROVAL

This study was conducted in accordance with Helsinki Declaration as revised in 2013. Statements of written informed consent from legally authorized representatives/parents are available. All figures and tables are sourced from authors’ clinical cases at IRCCS Azienda Ospedaliero‐Universitaria Sant’Orsola‐Malpighi di Bologna.

## 6 AUTHOR CONTRIBUTIONS

Dr Biffi and Dr Ziacchi performed devices implantation. Dr Piemontese, Dr Biffi, Dr Ziacchi wrote the manuscript. All the authors equally contributed to the data collection and analysis at the implant and during the patients' follow‐up. Dr Massaro was the language reviewer.

## Data Availability

All figures and tables are sourced from authors’ clinical cases at IRCCS Azienda Ospedaliero‐Universitaria Sant’Orsola‐Malpighi di Bologna.
